# Ionic Liquid as Surfactant Agent of Hydrotalcite: Influence on the Final Properties of Polycaprolactone Matrix

**DOI:** 10.3390/polym10010044

**Published:** 2018-01-05

**Authors:** Luanda Chaves Lins, Valeria Bugatti, Sébastien Livi, Giuliana Gorrasi

**Affiliations:** 1 University of Lyon, F-69003 Lyon, France; luandaqmc@gmail.com (L.C.L.); sebastien.livi@insa-lyon.fr (S.L.); 2 UMR 5223, Department of Engineering of Polymeric Materials, INSA Lyon, CNRS, F-69621 Villeurbanne, France; 3 Department of Industrial Engineering, University of Salerno-via Giovanni Paolo II, 132, 84084 Fisciano (Salerno), Italy; vbugatti@unisa.it

**Keywords:** polycaprolactone, layered double hydroxides, ionic liquids

## Abstract

This paper reports the surface treatment of layered double hydroxide (LDH) by using ionic liquid (IL) composed of phosphonium cation combined with 2-ethylhexanoate (EHT) counter anion as surfactant agent. Then, different amounts (1, 3, 5 and 7 wt %) of thermally stable organically modified LDH (up to 350 °C) denoted LDH-EHT were incorporated into polycaprolactone (PCL) matrix by mechanical milling. The influence of LDH-EHT loading has been investigated on the physical properties, such as the thermal and barrier properties, as well as the morphologies of the resulting nanocomposites. Thus, intercalated or microcomposite morphologies were obtained depending on the LDH-EHT loading, leading to significant reduction of the diffusion coefficient respect to water vapor. The modulation of barrier properties, using low functionalized filler amount, is a very important aspect for materials in packaging applications.

## 1. Introduction

In the last decade, layered double hydroxides (LDHs) have received great attention from industry and academia. Their structure is similar to that of brucite where Mg atoms are octahedrally coordinated to six oxygen atoms belonging to six groups of –OH; each –OH group is, in turn, shared by three octahedral cations and points the hydrogen atom to the interlayer space. When cations of Mg(II) are isomorphously replaced by cations of Al(III), the substitution generates positive charges that are counterbalanced by the presence of counter ions, generally CO_3_^2−^, Cl^−^, and NO_3_^−^, located in the interlamellar regions. The possibility to substitute these anions by ionic exchange procedures makes the hydrotalcites ideal solids to be used as host of potentially active molecules having a negative charge. LDHs have high level of purity and can be produced using simple procedures. They have been used as active filler for polymers and biopolymers for their capability of hosting active molecules [1,2,3,4,5,6,7], as catalysts for reactions [8,9], for the synthesis of polymers [10] and as surfactant adsorbents [11]. The exchange of the counter ions between the layers is not easy, therefore the modification of carbonated LDHs is generally carried out by calcination followed by immersion in a medium containing anions to be intercalated [12,13]. Very recently, ionic liquids (ILs) are attracting interest from several areas of basic and applied sciences, including chemistry, materials science, chemical engineering, and environmental science [14]. They are organic salts with low melting points (below 100 °C) based mainly on ammonium, imidazolium, pyridinium or phosphonium ILs combined with different counter anions such as halides, tetrafluoroborate (BF_4_^−^), hexafluorophosphate (PF_6_^−^), bistriflamide (TFSA), etc. ILs are considered as potentially environmentally friendly solvents having low volatility, low flammability, good chemical and thermal stabilities, and compatibility with organic and inorganic materials [15]. These characteristics make them a class of very versatile compounds. In fact, they have been used for organic synthesis [16], bio-processing operations, catalysis and gas separation [17], and inhibitors for the corrosion of magnesium alloys [18]. Recently, ILs have also been proposed as plasticizers, lubricants, structuring agents, or compatibilizing agents in several polymers [19,20,21,22,23,24]. They can also be used to overcome the low thermal stability of ammonium salts commonly used as organic modifiers of layered silicates, such as montmorillonites, that are used to enhance compatibility and dispersion of inorganic phases in organic media [25,26,27,28]. The hybrid organic-ionic nature of ILs and the ability to control their hydrophilic and lipophilic counterparts through proper selection of the constituent ions give rise to a complex set of phenomena, creating an area of study that is both fascinating and challenging. While attention has been devoted to ILs based on quaternary nitrogen, studies related to ILs based on quaternary phosphorous cations are still few. It has been demonstrated that some phosphonium based ILs have better properties than nitrogen-based ILs, [29,30]. In fact, research on nanocomposites containing clay minerals modified with phosphonium based ILs has recently increased [31,32,33]. In 2013, Bugatti et al. have developed a new method to deposit ionic liquid-modified LDHs on PLA films previously treated by plasma treatment leading to an increase of the water barrier properties of 30%. Recently, Kredatusova et al. have worked on the surface treatment of LDH by phosphonium ILs and they have used an environmentally friendly route based on microwave irradiation. Thus, they have demonstrated that the use of phosphonium ILs as surfactant agents of LDH induced an exfoliation morphology in polycaprolactone matrix. More recently, other authors have demonstrated that the incorporation of LDH-ILs into poly(butylene adipate-*co*-terephthalate) matrix led to a significant increase of its mechanical performances, especially of the stiffness without reducing the fracture behavior of the polymer matrix [34].

In a previous paper, we incorporated the LDH modified with trihexyl(tetradecyl)phosphonium 2-ethylhexanoate into a pectin matrix [35] for potential in biodegradable food packaging applications. Pectins are natural materials with no melting point, but high degradation temperature, and with high brittleness and poor elongation at break. These properties make them unable to be applied in flexible packaging. Poly(ε-caprolactone) (PCL) being a biodegradable polyester with low melting point and very high elongation at break but low elastic modulus appears one of the best candidate to be blended with pectins. The compatibilization between the two phases can be enhanced by the very wide chemistry of ionic liquids; in addition, also LDH can be considered an interesting filler hosting either ILs, either active molecules with peculiar functionalities. The aim of the present work is, then, to investigate the potential of layered double hydroxide modified with a phosphonium ionic liquid denoted trihexyltetradecylphosphonium 2-ethylhexanoate in PCL matrix for possible food packaging applications. In addition, the influence of the amount of modified LDHs will be evaluated on the morphologies but also in terms of thermal stability, surface analysis and barrier properties.

## 2. Experimental

### 2.1. Materials

Poly (ε-caprolactone) (PCL) Mn 80,000 was supplied from Sigma Aldrich (Milano, Italy). It was reduced in powder form in a mechanical mixer in presence of liquid nitrogen. A LDH (aluminum magnesium hydroxy carbonate), denoted PURAL^®^ MG 63 HT, was chosen as pristine anionic clay and was provided by Sasol Performance Chemicals (Hamburg, Germany). The IL, coded as IL-EHT, based on trihexyltetradecylphosphonium cation associated with 2-ethylhexanoate counter anion was kindly provided by Cytec Industries Inc. (Thorold, ON, Canada).

### 2.2. LDH-Ionic Liquid Preparation

According to the literature, two steps are required: (i) calcination of the pristine LDH at 500 °C for 24 h; and (ii) LDH and 2 AEC (anion exchange capacity) of phosphonium ILs dispersion in 200 mL of deionized water/tetrahydrofuran (THF) mixture (300/100 mL) [33,36,37]. The suspensions were stirred and mixed at 60 °C for 24 h. The resulting precipitate was filtered and washed 5 times with THF. The residual solvent was removed by evaporation under vacuum and finally, the treated LDH was dried overnight at 80 °C. From the thermogravimetric analysis TGA (see inset of Figure 4) the amount of physisorbed and intercalated water (25–220 °C) is ≅15 wt %, the amount of carbonate anion and IL (250–500 °C) is ≅25 wt % and the amount of LDH (>500 °C) into the LDH-EHT is ≅60 wt %. The phosphonium ionic liquid used for the surface treatment and the following abbreviation used to designate the LDH-IL are summarized in Table 1.

### 2.3. Composite Films Preparation

The incorporation of the filler into PCL was achieved by High Energy Ball Milling (HEBM) method. Powders composed of PCL and filler (vacuum dried for 24 h) were milled at different filler loading (i.e., 1, 3, 5, and 7 wt %) at room temperature in a Retsch (Haan, Germany) centrifugal ball mill (model S 100). The powders were milled in a cylindrical steel jar of 50 cm^3^ with 5 steel balls of 10 mm of diameter. The rotation speed used was 580 rpm. and the milling time was 60 min. The pure PCL, taken as reference, was milled in the same experimental conditions of the composites. The milled powders, were molded in a Carver laboratory press between two Teflon sheets, at 100 °C, followed by a quick cooling at ambient temperature. Films 150 µm thick were obtained and analyzed.

### 2.4. Methods of Investigation

*Wide-angle X-ray diffraction spectra (WAXD)* were collected on a Bruker D8 Advance X-ray diffractometer (Karlsruhe, Germany). A bent quartz monochromator was used to select the Cu Kα_1_ radiation (*k* = 0.15406 nm) and run under operating conditions of 45 mA and 33 kV in Bragg-Brentano geometry.

*Transmission electron microscopy (TEM)* was carried out using a Philips CM 120 field emission scanning electron microscope (Philips, Eindhoven, The Netherlands) with an accelerating voltage of 80 kV. The samples were cut using an ultramicrotome equipped with a diamond knife, to obtain 60-nm-thick ultrathin sections. Then, the sections were set on copper grids.

*Thermo Gravimetric Analysis (TGA)* was carried out with a Mettler TC-10 thermobalance (Novate Milanese, Italy) from 25 °C to 600 °C at a heating rate of 10 °C/min under an air flow.

*Differential scanning calorimetry (DSC)* were carried out using by means of a DTA Mettler Toledo (DSC 30, GmbH, Greifensee, Switzerland) under nitrogen atmosphere. The films were heated from 25 °C to 100 °C at a heating rate of 10 °C/min.

*Fourier transform infrared (FTIR)* absorption spectra were recorded by a Perkin-Elmer spectrometer (Bruker Italia, Milano, Italy), model Vertex 70 (average of 32 scans, at a resolution of 4 cm^−1^). Composite films, having the same thickness (≅150 μm) were analyzed. The LDH-P2 filler was analyzed in powder form after preparing a KBr based tablet (~1 mg of filler sample and ~100 mg of KBr).

*Diffusion coefficients* were evaluated, using the microgravimetric method, at different vapor activities (a = *P*/*P*_0_), where *P* is the actual pressure to which the sample was exposed, and *P*_0_ the saturation pressure at the test temperature. The penetrant was water vapor and the experiments were conducted at 30 °C. The transport properties were measured with water vapor in a range of activities from 0.2 to 0.6. Measuring the increase of weight with time for the samples exposed to the vapor at a given partial pressure, *P*, both the equilibrium value of the sorbed vapor, *C*_eq_ (g/100 g), and the diffusion coefficient, *D*, were obtained.

*Surface energies* were determined with the sessile drop method using a Contact Angle System OCA, Dataphysics^®^ (DataPhysics Instruments GmbH, Filderstadt, Germany). From contact angle measurements with water and diiodomethane as test liquids, the polar and dispersive components of surface energy were determined using the Owens-Wendt theory [38].

## 3. Results and Discussion

### 3.1. Morphologies of PCL/LDH Nanocomposites

**Figure 1:**
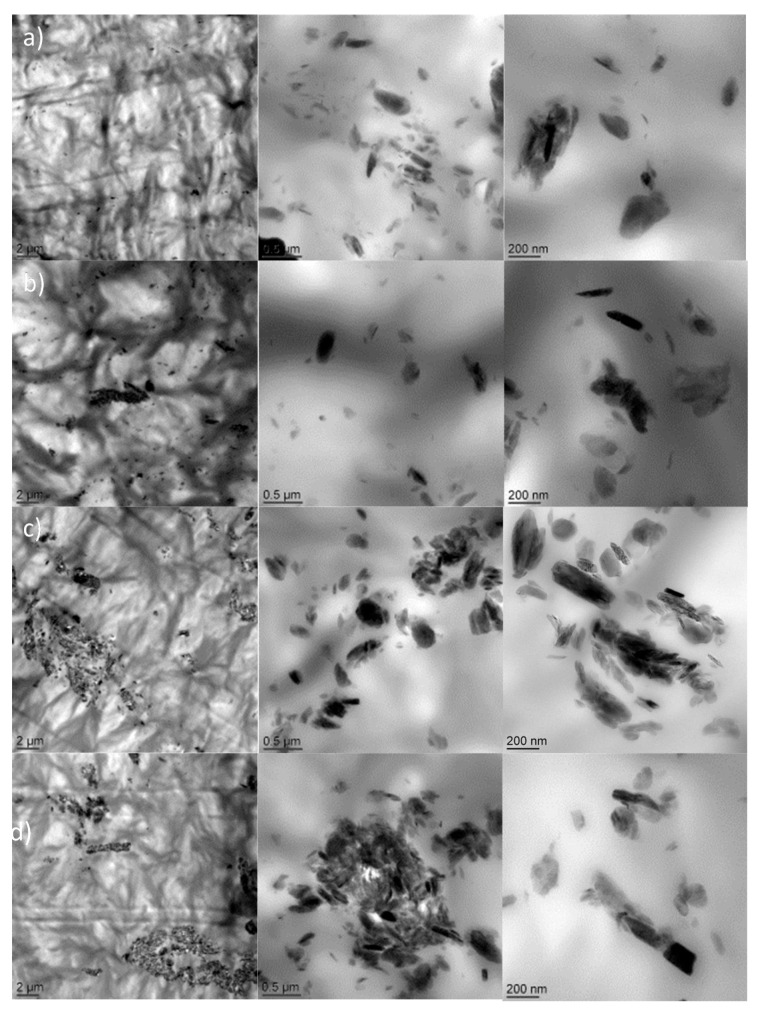
TEM micrographs of: (**a**) PCL + 1% LDH-EHT; (**b**) PCL + 3% LDH-EHT; (**c**) PCL + 5% LDH-EHT; and (**d**) PCL + 7% LDH-EHT.

Figure 1 shows the TEM micrographs evaluated on all composites. Two types of morphologies have been observed. For PCL containing 1 and 3 wt % of LDH-EHT, a homogenous dispersion of LDH has been obtained with the presence of small tactoïds. When enlargements at 500 and 200 nm have been performed, the majority of tactoïds have sizes less than 500 nm combined with the presence of few individual platelets. In the opposite, an increase of the amount of LDH-EHT (5 and 7 wt %) induced the formation of agglomerates but also a less homogeneous dispersion. In both cases, a microcomposite morphology is obtained for PCL containing 1, 3, 5 and 7 wt % of LDH-EHT.

**Figure 2:**
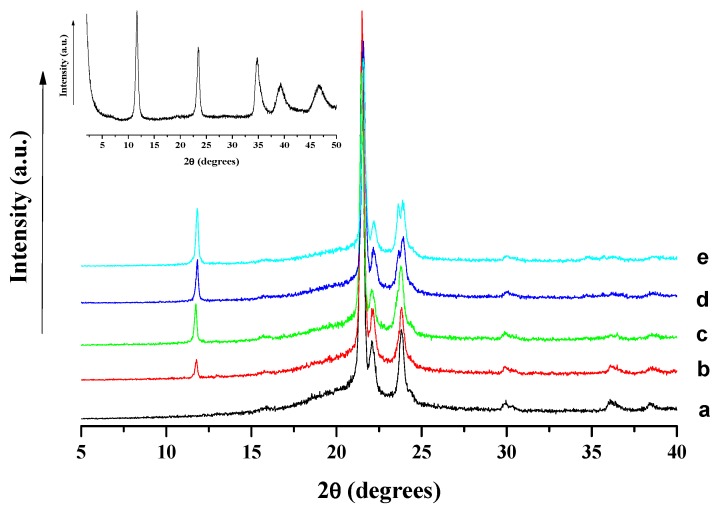
XRD spectra of: PCL (**a**); PCL/1% LDH-EHT (**b**); PCL/3% LDH-EHT (**c**); PCL/5% LDH-EHT (**d**); and PCL/7% LDH-EHT (**e**). Inset reports the XRD spectrum of LDH-EHT.

Figure 2 reports the XRD spectra of PCL film and composites filled with LDH-EHT at different loading. The inset reports the spectrum of the LDH-EHT. In a previous work, we have observed by XRD that the surface treatment of LDH by phosphonium ILs highlighted only one diffraction peak at 2θ = 11.6° corresponding to a basal spacing of 7.7 Å [33]. This result showed no influence of the intercalation of IL between clay layers and corresponded to the basal spacing of the pristine LDH. According to the literature, the absorption of the carbonate anions takes place during the surface treatment [33]. In addition, these results can be explained by the presence of short alkyl chains (<12 carbons) on the anion leading to a planar configuration such as monolayer type. Indeed, Lagaly and Weiss have demonstrated on MMT that the surfactant chain length plays a key role on the different arrangements of organic salts between the clay layers [39,40]. In this work, the 2-ethylhexanoate counter anion is only composed of six carbons and one carbonyl bond (C=O). The carbonate anion also has only one carbonyl bond, and if IL-EHT assumes a planar configuration, no difference can be observed by XRD. The neat PCL matrix and the resulting nanocomposites containing different amounts of LDH-EHT i.e., 1, 3, 5 and 7 wt % exhibit different diffraction peaks. In fact, the diffraction peaks observed at 2θ = 21.2°, 21.8° and 23.5°, corresponding to basal spacing of 4.2 Å, 4.1Å, and 3.8 Å, respectively, are attributed to the crystallinity of the polycaprolactone, whereas the diffraction peak observed at 2θ = 11.6° is characteristic of the basal spacing of the pristine LDH, suggesting an aggregation of the LDH-EHT into the polymer matrix. These results clearly confirm the morphologies obtained by transmission electron microscopy (Figure 1).

**Figure 3:**
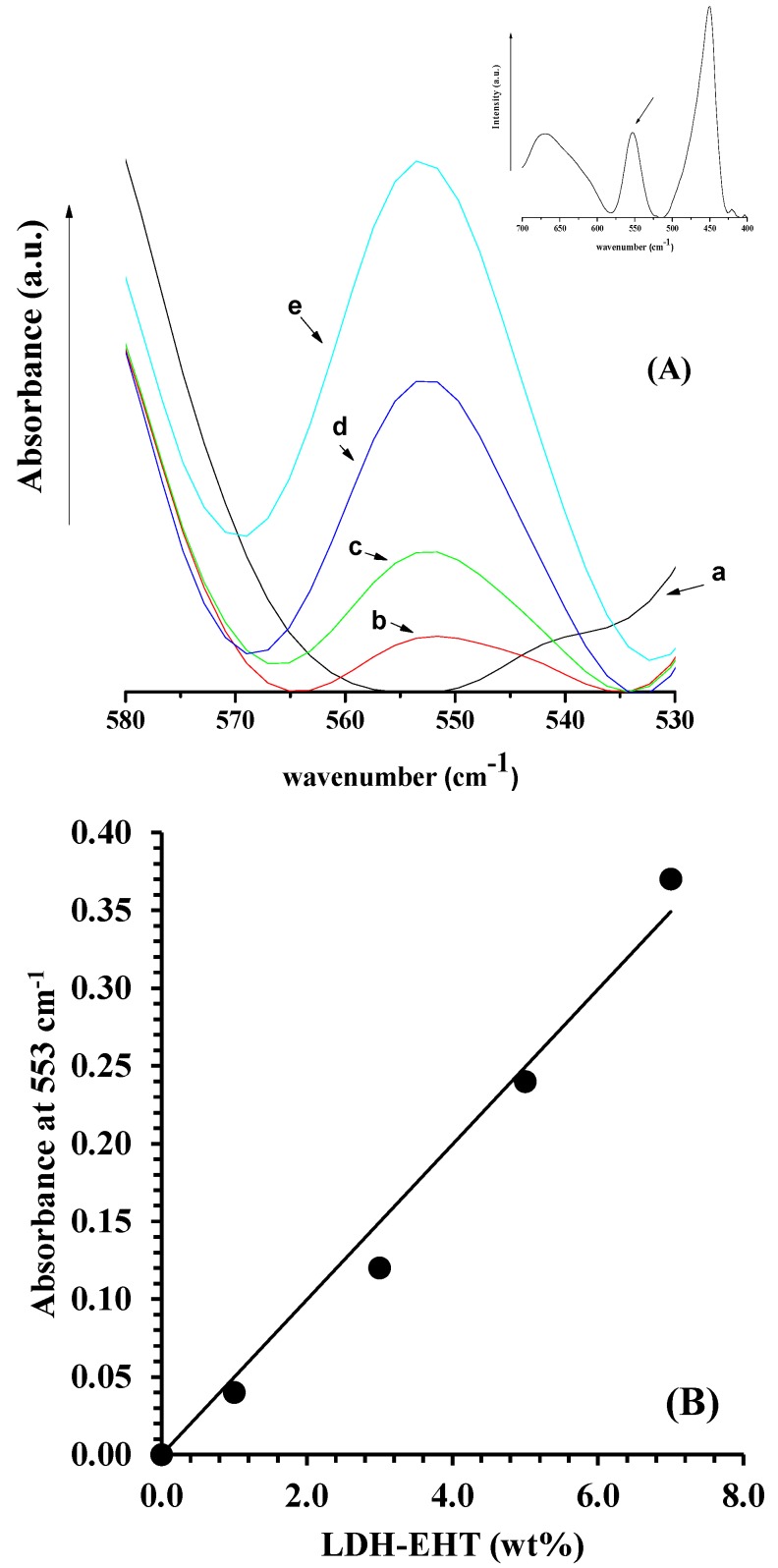
(**A**) FTIR spectra, in the range 530–580 cm^−1^, of: PCL (a); PCL/1% LDH-EHT (b); PCL/3% LDH-EHT (c); PCL/5% LDH-EHT (d); and PCL/7% LDH-EHT (e). The inset reports the spectrum of pure LDH-EHT. (**B**) Absorbance at 553 cm^−1^ as a function of LDH-EHT (wt %).

Figure 3A reports the FTIR spectra, in the range 530–580 cm^−1^, of PCL and composites at different filler loading, evaluated on films having the same thickness (150 μm). The inset reports the spectrum of pure LDH-EHT. In this range, PCL does not show any peak, while, for LDH, the band at 553 cm^−1^ is assigned to translation modes of the hydroxyl groups mainly influenced by the trivalent aluminum [41]. Figure 3B reports the absorbance at 553 cm^−1^ as function of filler amount. It is evident that the absorbance at 553 cm^−1^ is linear with the filler loading, making this technique a useful tool for quantitative analysis.

### 3.2. Thermal Properties of PCL/LDH Nanocomposites

**Figure 4:**
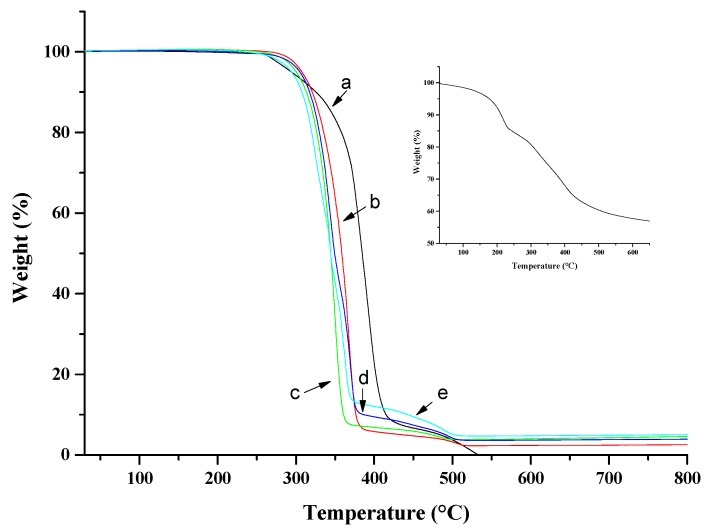
TGA analysis in air evaluated: PCL (**a**); PCL/1% LDH-EHT (**b**); PCL/3% LDH-EHT (**c**); PCL/5% LDH-EHT (**d**); and PCL/7% LDH-EHT (**e**). The inset reports the TGA on LDH-EHT.

Figure 4 reports the TGA analysis evaluated on all the PCL composites. PCL is also reported for comparison. The inset of Figure 4 reports the TGA of the hybrid LDH-EHT.

The use of IL-EHT as surfactant agent of LDH generated different TGA profile of pristine LDH with the formation of two degradation peaks at 317 °C and 398 °C compared to 310 °C and 430 °C for LDH intercalated by carbonate counter anion. These results suggested the combined presence of carbonate and 2-ethylhexanoate counter anions which confirm XRD results. These results are consistent with the study where Kredatusova et al. showed by FTIR and XPS the presence of this IL onto the clay surface but also into the clay layers [33].

The degradation of PCL occurs in two steps: The first one implies a statistical rupture of the polyester chains via ester pyrolysis reaction with production of 5-hexenoic acid, H_2_O and CO_2_. The second step leads to the formation of ε-caprolactone (cyclic monomer) as result of an unzipping depolymerization process [42]. The first degradation step is slightly anticipated in the composites, and the second step occurs around the temperature of degradation of unfilled PCL. This can be due either to the presence of LDH-EHT which starts to decompose at a lower temperature, or to the decrease of molecular weight of PCL for the milling treatment [43]. To better demonstrate this assumption, we submitted the PCL to the same milling processing of the composites and evaluated the Mw, by GPC analysis. We found a decreasing of Mw from 195 KDa of the unmilled PCL to 120 KDa of milled material. Table 2 reports the *T*_10%_ and *T*_50%_ of weight loss for all samples. In addition, different authors have demonstrated that the presence of water between LDH layers combined to the formation of metal oxides during the heating of hydrotalcite could accelerate the degradation of the polymer matrix [44,45]. In addition, the low thermal stability of the IL-EHT physisorbed on the LDH (around 340 °C) can also be explained an advanced degradation of the polymer matrix and highlighted by Xanthos et al. on a PLA matrix [46].

### 3.3. Surface Analysis and Transport Properties of PCL/LDH Nanocomposites

According to the literature, it is well-known that the presence of ionic liquids into polymer matrix has a significant influence on the hydrophobic behavior of the polymer materials [21,24]. In a previous work, Livi et al. have demonstrated that the incorporation of only 2 wt % of ILs into PBAT matrix generated a significant increase of the water barrier properties due to the hydrophobic nature of phosphonium ionic liquids determined by Coutinho et al. [47,48]. In other works, the same authors have also demonstrated that the use of ILs as additives of epoxy networks induced a significant decrease of the polar component leading to more hydrophobic networks [49]. Thus, to determine the impact of the LDH-EHT on the surface energies of the polymer nanocomposites, the contact angles and surface energy on the neat polycaprolactone and the resulting nanocomposites containing 1, 3, 5 and 7 wt % of LDH-EHT determined by the sessile drop method are summarized in Table 3. Whatever the amount of LDH-EHT introduced into PCL matrix, similar values of surface energies were obtained between 30.8 and 33.5 mN/m. However, differences were observed concerning the values of the polar components. In fact, the incorporation of 1 and 3 wt % of organically modified LDH induced decreases in the polar component from 5 mN/m (neat PCL) to 0.4 mN/m (1 wt % LDH-EHT) and 0.8 mN/m (3 wt % LDH-EHT). These results confirm the presence of IL-EHT in the surface of LDHs and a homogeneous dispersion of the LDH-EHT, as seen on TEM micrographs (Figure 1). According to the literature, this decrease of the polar component is explained by the hydrophobic nature of IL-EHT functionalized by long alkyl chains [49,50]. Thus, different authors have demonstrated that the use of phosphonium IL as additives or surfactant agents of layered silicates such as montmorillonite (MMT) induced a significant reduction of the polar component [24]. Then, for PCL containing 5 and 7 wt % of the LDH-EHT, an increase in the polar component is observed (0.4 mN/m to 3.3 mN/m). These results can be explained by an increase of the amount of LDH-EHT. In fact, we have previously demonstrated by TGA that LDH-EHT contained 15 wt % of physisorbed and intercalated water. Consequently, an increase in the modified clays resulted in a slight decrease in the hydrophobicity of the PCL matrix through the polar component.

**Figure 5:**
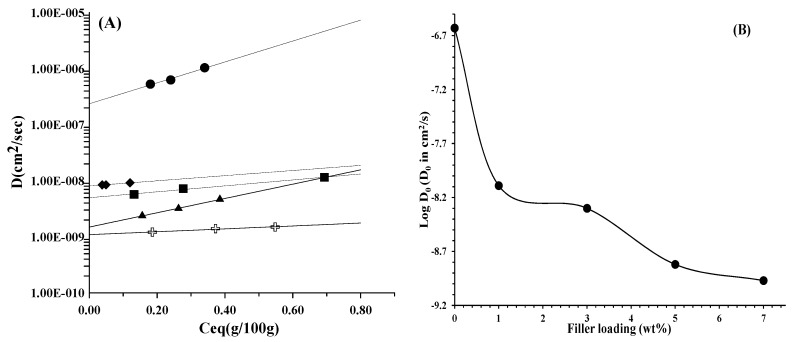
(**A**) The diffusion coefficient, *D* (cm^2^/s), as function of *C*_eq_ (g/100 g) of water vapor for: PCL (●); PCL/1% LDH-EHT (◆); PCL/3% LDH-EHT (■); PCL/5% LDH-EHT (▲); and PCL/7% LDH-EHT (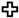); and (**B**) the log *D*_0_ as function of filler (LDH-EHT) loading (wt %).

The transport phenomena of small molecular weight molecules, such as water vapor, through polymeric multiphase systems are strictly influenced by the texture of the materials. In the case of composites, the dispersed phase is expected to play a significant role for the diffusion of the penetrating molecules [26]. Figure 5A reports the diffusion coefficient, *D* (cm^2^/s), as function of *C*_eq_ (g/100 g) of water vapour for PCL and the analyzed composites. A linear dependence of the diffusion with respect the equilibrium water sorption can be observed for all samples. It is, then, possible to extrapolate the thermodynamic diffusion coefficient, *D*_0_, using the following empirical equation:

(1) $$D\;=\;D_{0}\;\exp\;\left(\gamma \;C_{\mathrm{eq}}\right)$$

The thermodynamic diffusion coefficient is related to the fractional free volume and the tortuosity of the pathway. *D*_0_ was extrapolated for all the samples according to Equation (1). The log *D*_0_ versus filler loading is reported in Figure 5B. It is evident a significant decrease of the thermodynamic diffusion coefficient, even with 1 wt % of filler, a smooth decreasing going from 1 wt % to 3 wt % and a quite linear decreasing up to 7 wt % of filler loading. In XRD analysis, it was observed that the crystalline form of PCL is the same of the composites, and the degree of crystallinity, evaluated by DSC analysis, and here not reported is almost the same in unfilled sample and composites and ranges between 47% and 52%. The decreasing of the diffusion cannot be ascribed, then, to an increasing of crystallinity degree. The presence of the filler increases to a large extent the tortuosity of the pathway, leading to a decreasing in the diffusion. It is worth noticing that, already at 1 wt % of filler, the diffusion decreases of more than one order of magnitude. We have also attributed these results to the presence of IL into LDH. In fact, Coutinho et al. have investigated the effect of water on imidazolium and phosphonium ionic liquids combined with different counter anions [48,51]. For a small amount of water, the authors have highlighted a migration of the water to the near surface of IL inducing the formation of hydrogen bonding’s with the counter anion. Oppositely, the use of extra water led to the formation of water clusters generating an increase of the surface tension of the neat ILs. By sessile drop method, we have observed an increase of the polar component from 3 wt % of LDH-EHT incorporated in the PCL matrix. Based on these different results and the obtained diffusion coefficients, we can suggest that the presence of only 1 wt % of IL modified-LDH in the polymer matrix can capture/slow water molecules through hydrogen-bonding interactions with the counter anion of IL-EHT. However, an increase in the amount of LDH-EHT containing up to 15 wt % of water generates a progressive formation of water clusters thus reducing the effect of the ionic liquid in favor of the dispersion of LDH, especially from 3 wt % to 5 wt % where the formation of well-dispersed aggregates into PCL matrix helps to increase the tortuosity of the pathway.

## 4. Concluding Remarks

In this work, thermally stable organically modified hydrotalcite were prepared by using one phosphonium ionic liquid as modifier agent and were used as nanoparticles in PCL matrix. Then, different amounts of the LDH-EHT (1, 3, 5, 7 wt %) were introduced and their influence was investigated on the morphologies and the final properties of the polymer matrix. Two tendencies were observed in function of the quantity of LDH-EHT. For PCL containing 1 and 3 wt % of treated LDH, a homogeneous dispersion was obtained with the presence of small tactoïds leading to a significant decrease of the diffusion coefficient of water vapor, as well as a significant decrease of the polar component from 5 mN/m to 0.4 mN/m. Oppositely, an increase in the amount of LDH induced a microcomposite morphology characterized by the formation of large agglomerates. Moreover, whatever the amount of LDH-EHT incorporated into PCL matrix, a reduction of the thermal stability of the PCL nanocomposites was demonstrated. In summary, the use of small quantities of LDH has a significant impact on the water barrier properties of the polymer matrix opening up new potential prospects in food packaging.

## Figures and Tables

**Table 1:** Designation of the phosphonium ionic liquid (IL) used for the modification of LDH.

Ionic Liquid	Chemical Structure	Code
Trihexyl(tetradecyl)phosphonium 2-ethylhexanoate	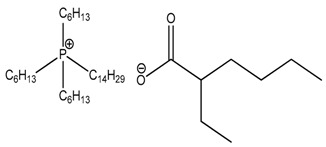	EHT

**Table 2:** Temperature at 10% and 50% of weight loss, extracted from TGA analysis (Figure 4).

Sample	*T*_10%weight loss_ (°C)	*T*_50%weight loss_ (°C)
**PCL**	327	385
**PCL + 1% LDH-EHT**	320	359
**PCL + 3% LDH-EHT**	315	343
**PCL + 5% LDH-EHT**	317	348
**PCL + 7% LDH-EHT**	307	344

**Table 3:** Polar and dispersive components of the surface energy on the neat PCL and the resulting nanocomposites, from contact angles with water and diiodomethane at room temperature.

Sample	*θ*_water_ (°)	*θ*_CH_2_I_2__ (°)	Surface Energy (mN/m)	Dispersive Component (mN/m)	Polar Component (mN/m)
**PCL**	77 ± 3	62 ± 0.4	30.8	26.1	4.7
**PCL + 1% LDH-EHT**	113 ± 11	62 ± 2.4	31.6	31.2	0.4
**PCL + 3% LDH-EHT**	106 ± 3	52 ± 1.8	33.1	32.3	0.8
**PCL + 5% LDH-EHT**	87 ± 4	49 ± 0.5	33.2	31.0	2.2
**PCL + 7% LDH-EHT**	85 ± 3	50 ± 3.6	33.5	30.2	3.3
